# Neurogenic Defects Occur in *LRIG2*-Associated Urinary Bladder Disease

**DOI:** 10.1016/j.ekir.2023.04.017

**Published:** 2023-04-30

**Authors:** Celine Grenier, Filipa M. Lopes, Anna M. Cueto-González, Eulàlia Rovira-Moreno, Romy Gander, Benjamin W. Jarvis, Karen D. McCloskey, Alison M. Gurney, Glenda M. Beaman, William G. Newman, Adrian S. Woolf, Neil A. Roberts

**Affiliations:** 1 Division of Cell Matrix Biology and Regenerative Medicine, School of Biological Sciences, Faculty of Biology Medicine and Health, University of Manchester, Manchester, UK; 2 Department of Clinical and Molecular Genetics, Vall d'Hebron Barcelona Hospital Campus, Catalonia, Spain; 3 Medicine Genetics Group, Vall Hebron Research Institute, Vall d'Hebron Barcelona Hospital Campus, Autonomous University of Barcelona, Barcelona, Spain; 4 Department of Pediatric Surgery, Pediatric Urology and Renal Transplant Unit, University Hospital Vall D'Hebron Barcelona, Hospital Vall D'Hebron, Barcelona, Spain; 5 Patrick G. Johnston Center for Cancer Research, School of Medicine, Dentistry and Biomedical Sciences, Queen’s University Belfast, Belfast, UK; 6 Division of Pharmacy and Optometry, School of Health Sciences, Faculty of Biology Medicine and Health, University of Manchester, Manchester, UK; 7 Manchester Center for Genomic Medicine, Manchester University NHS Foundation Trust, Manchester Academic Health Science Center, Manchester, UK; 8 Division of Evolution, Infection and Genomics, Faculty of Biology, Medicine and Human Sciences, University of Manchester, Manchester, UK; 9 Royal Manchester Children's Hospital, Manchester University NHS Foundation Trust, Manchester Academic Health Science Center, Manchester, UK

**Keywords:** bladder, LRIG2, neurogenic, Ochoa, syndrome, urofacial

## Abstract

**Introduction:**

Urofacial, or Ochoa, syndrome (UFS) is an autosomal recessive disease featuring a dyssynergic bladder with detrusor smooth muscle contracting against an undilated outflow tract. It also features an abnormal grimace. Half of individuals with UFS carry biallelic variants in *HPSE2*, whereas other rare families carry variants in *LRIG2.LRIG2* is immunodetected in pelvic ganglia sending autonomic axons into the bladder. Moreover, *Lrig2* mutant mice have abnormal urination and abnormally patterned bladder nerves. We hypothesized that peripheral neurogenic defects underlie *LRIG2*-associated bladder dysfunction.

**Methods:**

We describe a new family with *LRIG2*-associated UFS and studied *Lrig2* homozygous mutant mice with *ex vivo* physiological analyses.

**Results:**

The index case presented antenatally with urinary tract (UT) dilatation, and postnatally had urosepsis and functional bladder outlet obstruction. He had the grimace that, together with UT disease, characterizes UFS. Although *HPSE2* sequencing was normal, he carried a homozygous, predicted pathogenic, *LRIG2* stop variant (c.1939C>T; p.Arg647∗). *Lrig2* mutant mice had enlarged bladders. *Ex vivo* physiology experiments showed neurogenic smooth muscle relaxation defects in the outflow tract, containing the urethra adjoining the bladder, and in detrusor contractility. Moreover, there were nuanced differences in physiological outflow tract defects between the sexes.

**Conclusion:**

Putting this family in the context of all reported UT disease-associated *LRIG2* variants, the full UFS phenotype occurs with biallelic stop or frameshift variants, but missense variants lead to bladder-limited disease. Our murine observations support the hypothesis that UFS is a genetic autonomic neuropathy of the bladder affecting outflow tract and bladder body function.

## Graphical abstract

**Figure:**
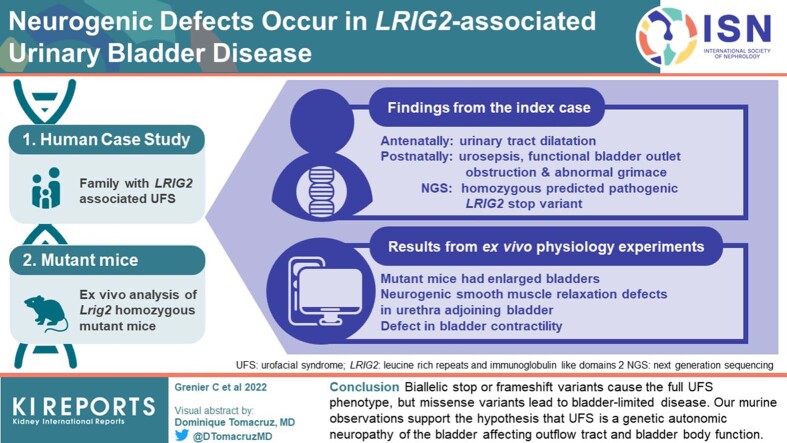

The mammalian UT comprises the kidneys, ureters, bladder, and urethra. Congenital UT malformations (UTMs) occur in 4 per 1000 human births^1^^,^^2^ and UTMs account for approximately half of all children with severe kidney failure.^3^ An estimated fifth of young adults with severe kidney failure were born with UTMs.^4^ Some individuals with a UTM have defined monogenic causes,^5^^,^^6^ and although most genetic research has focused on kidney malformations, monogenic causes of lower UTMs are now being discovered.^7^, ^8^, ^9^, ^10^, ^11^

UFS is an autosomal recessive condition causing lifelong ill health with potentially fatal complications.^12^^,^^13^ Affected females and males have dyssynergic bladders in which the detrusor smooth muscle in the bladder body contracts against an outflow tract that fails to dilate fully. Therefore, individuals with UFS experience frequent but incomplete urinary voiding. This functional bladder outflow obstruction leads to pooling of urine under high hydrostatic pressure within the bladder predisposing to vesicoureteric reflux, urosepsis, pyelonephritis, and kidney failure.^12^^,^^13^ These UT features are accompanied by a characteristic grimace on smiling, hence the term “urofacial.”^12^

As reviewed,^11^^,^^14^ about half of genetically tested UFS individuals carry biallelic variants in *HPSE2* (OMIM UFS2 #236730), which encodes heparanase-2, a protein that binds heparan sulfate and also inhibits the endoglycosidase enzymatic activity of a related protein called heparanase.^15^^,^^16^

Not all individuals with UFS have *HPSE2* variants. A small number of individuals with UFS have been reported to carry biallelic variants of *LRIG2* (OMIM USF2 #615112).^17^^,^^18^ *LRIG2* codes for leucine rich repeats and immunoglobulin-like domains 2, 1 of 3 mammalian LRIG proteins.^19^ Homozygous *Lrig2* or *Hpse2* mutant mice manifest aberrant urinary voiding,^20^^,^^21^ making them an appropriate model to study UFS in the laboratory.

We report here a new family with UFS, associated with a homozygous *LRIG2* variant, and place this in the context of all other UT disease-associated *LRIG2* variants reported to date. We hypothesized that peripheral neurogenic defects underlie *LRIG2*-associated bladder dysfunction. Accordingly, we studied *Lrig2* mutant mice with a battery of *ex vivo* physiology analyses. Our murine observations support the hypothesis that UFS is a genetic, autonomic neuropathy of the bladder with functional defects in both the outflow tract and detrusor smooth muscle in the bladder body.

## Methods

### Human Studies

The family described below was assessed at the Department of Clinical and Molecular Genetics and the Department of Pediatric Surgery, Pediatric Urology and Renal Transplant Unit, Hospital Vall D´Hebron, Barcelona, Spain. The parents, legal guardians, gave written informed consent, as approved by Vall d'Hebron Hospital Ethics Committee, for molecular genetic testing. The family also gave permission for their clinical story to be told, and informed consent for their child’s face to be shown here.

### Mutant Mice

*Lrig2* mice carrying a targeted deletion of exon 12^21^^,^^22^ were maintained on a C57BL/6 background in the Biological Services Facility of The University of Manchester (Home Office Project License PP1688221). Homozygous wild-type (WT) and homozygous *Lrig2* (Mut) mice were generated by mating heterozygous parents. Lrig2 protein is present in normal mouse bladders but is not immunodetected in Mut bladders.^21^

### *Ex Vivo* Physiology on Isolated Bladder Tissues

WT and *Lrig2* Mut mice were culled by cervical dislocation in accordance with Schedule 1 of the Animals (Scientific Procedures) Act 1986 (ASPA) and with local Animal Welfare Ethical Review Body (AWERB) approval (University of Manchester). Experiments were performed on bladder preparations from WT and Mut mice as described previously for *Hpse2* mutant mice.^23^ Briefly, the detrusor and outflow tracts were separated and mounted in myograph chambers (Danish Myo Technology, Hinnerup, Denmark) to measure isometric tension in physiological solution maintained at 37 °C. Detailed protocols are described in the Supplementary Methods. Relaxation of the outflow tract is mediated by nitrergic nerves.^23^, ^24^, ^25^ Bladder outflows, containing part of the urethra nearest the bladder, were isolated by sharp dissection at the bladder neck and above the external sphincter. The tissue was cleaned of fat and any adherent nonoutflow tract tissues. Because incomplete dilatation of the bladder outflow tract is a key feature in people with UFS,^12^ the ability of *Lrig2* Mut outflow tracts to relax in response to electrical field stimulation (EFS; 0.5–15 Hz, 80 V, 1 ms) or the directly acting nitric oxide (NO) donor, sodium nitroprusside (SNP), were compared with WT littermates. Relaxation was measured against a background of preconstriction, which was evoked in males by the α-1 receptor agonist, phenylephrine (PE, 5 μM). Female outflow tracts contracted poorly to PE, confirming an earlier report.^26^ Relaxation was calculated by determining the absolute difference immediately before EFS application to the maximum decrease during stimulation, relative to the stable PE-induced increase in tension (i.e., the “PE baseline”). Because female mouse urethras express transcripts encoding the arginine vasopressin (AVP) V1a receptor,^27^ they were instead precontracted with (AVP). In separate experiments, outflow tracts or bladder bodies were stimulated with 50 mM KCl to directly depolarize the smooth muscle, which stimulates Ca^2+^ influx through voltage-gated Ca^2+^ channels to elicit contraction. Bladder bodies were subjected to EFS (0.5–25 Hz, 80 V, 1 ms) to induce frequency-dependent contractions mediated by acetylcholine-releasing parasympathetic nerves. Contraction was also evoked directly by the muscarinic agonist, carbachol (10 nM – 50 μM) and compared between WT and Mut mice.

### Histology

Detailed protocols are provided in the Supplementary Methods*.* Histologic evaluation of mouse bladder bodies was performed as previously described.^21^^,^^28^, ^29^, ^30^ We assessed anatomy by hematoxylin staining, collagen by picrosirius red staining, and F4/80 macrophages and transforming growth factor β1 (TGFβ1) by immunostaining. Alignment or coherence of collagen fibrils was also assessed.^31^

### Statistics

Details of data and statistical analysis are given in the Supplementary Methods.

## Results

### Family History

**Figure 1:**
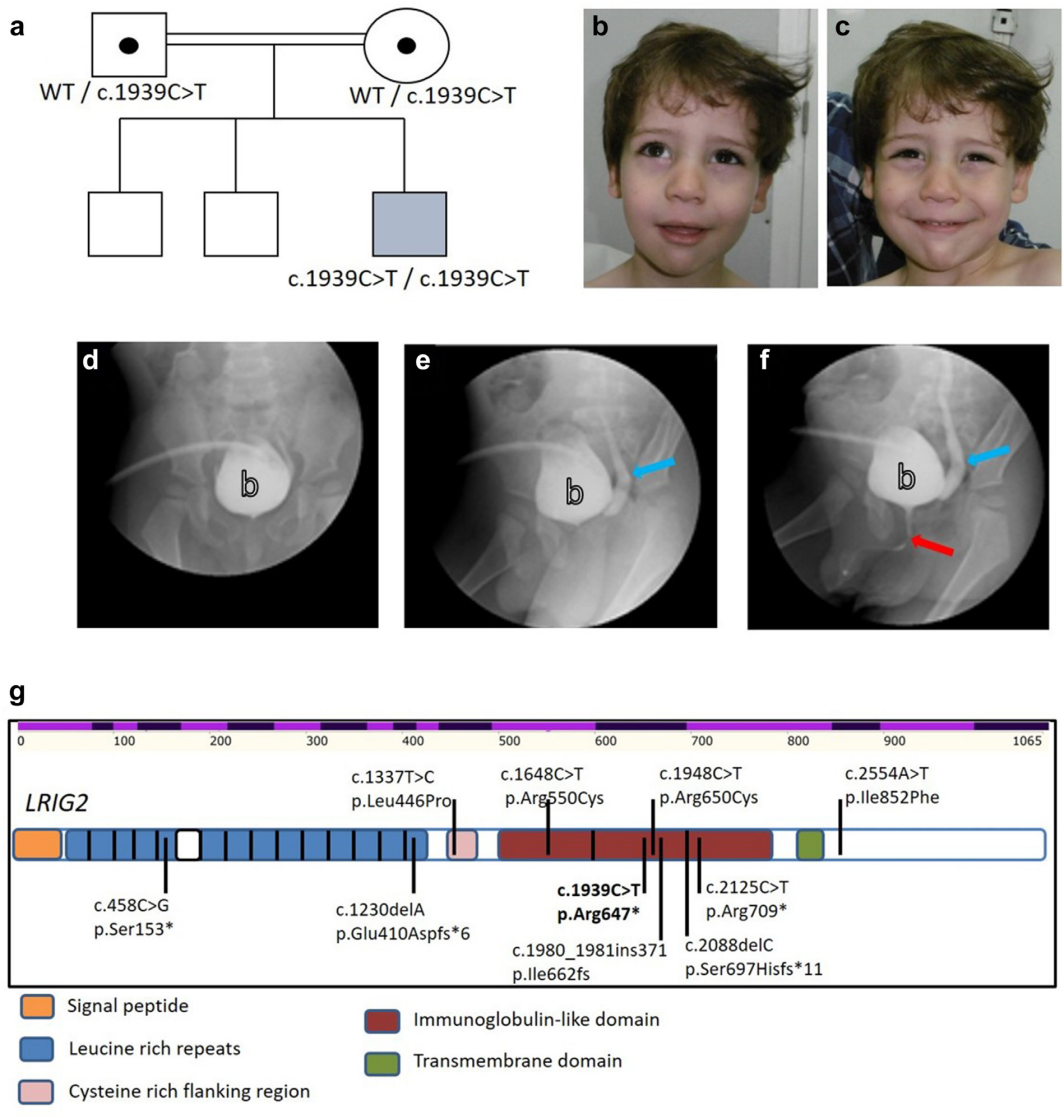
Identification of a novel pathogenic variant in *LRIG2* in a family affected by urofacial syndrome. (a) Pedigree of affected family showing the inheritance of the pathogenic variant. (b) and (c) Facial appearance of the affected child, before and during smiling. (d–f) Cystourethrogram sequential images as the bladder was filled *via* a suprapubic catheter, showing vesicoureteric reflux (blue arrow) and an anatomically patent urethra (blue arrow). (g) Location of all identified *LRIG2* pathogenic variants associated with UT disease. The uppermost alternating light and dark purple row indicates positions of *LRIG2* exons and base position. Below this, the protein is depicted with its key domains indicated in different colors. Missense variants are shown above the protein sequence, whereas nonsense and frameshift variants are shown below the protein. The variant described in this paper is shown in bold and is located in the part of the gene coding for an immunoglobulin-like domain. WT, wild-type.

The index case was a 6-year-old boy born to first cousin parents from Morocco (Figure 1a). He has a grimace on smiling, such that his eyes close and the corners of his mouth fail to elevate (Figure 1b and c). The pregnancy leading to his birth was complicated by gestational diabetes mellitus, and bilateral hydronephrosis was detected on fetal ultrasound, in the third trimester. At birth (full-term), weight and head circumference were within normal limits; however, increased body length was noted (55 cm, >99th percentile) and attributed to gestational diabetes. The first postnatal weeks were complicated by urosepsis, and ultrasound revealed bilateral ureterohydronephrosis with a thickened bladder wall. Cystography revealed vesicoureteral reflux; however, the urethra appeared normal, excluding posterior urethral valves (Figure 1d–f). A clinical diagnosis of functional bladder outlet obstruction was made, and the bladder was drained by a percutaneous cystostomy device. Assessed at 6 years, his psychomotor, language, and learning development were normal, as were weight, height, and head circumference. His plasma creatinine concentration was 0.41 mg/dl (36 micromol/l), within the normal range (0.24–0.73 mg/dl). *HPSE2* and *LRIG2* were sequenced (directed sequencing, Next Generation Sequencing). No variants were found in *HPSE2* but a homozygous variant (NM_014813.2): c.1939C>T (p.Arg647∗), classified as pathogenic, according to American College of Medical Genetics guidelines,^32^ was identified in *LRIG2.* The stop codon was in exon 14 coding for an immunoglobulin-like domain (Figure 1g). The parents were healthy, heterozygous carriers. The index case has 2 older brothers who are clinically well; they have no urinary symptoms, lack a grimace on smiling, and have had normal abdominal ultrasound scans; their DNA is not available for testing. The variant in the index case is presented in the context of all other reported UT disease-associated variants in *LRIG2* in Figure 1g and Table 1.

**Table 1:** UT disease-associated *LRIG2* variants reported to date

Variant in *LRIG2* sequence	Predicted change in protein	Exon	Reference	Clinical features
c.458C>G	Stop variant p.Ser153∗	4	Sinha *et al.*^18^	UFS (grimace and urinary tract disease)
c.1230delA	Frameshift variant p.Glu410Aspfs∗6	10	Stuart *et al.*^17^	UFS (grimace and urinary tract disease)
c.1337T>C	Misssense variant p.Leu446Pro	12	Roberts *et al.*^21^	Urinary tract disease (without UFS grimace)
c.1648C>T	Misssense variant p.Arg550Cys	13	Stuart *et al.*^17^	Urinary tract disease (without UFS grimace)
c.1939C>T	Stop variant p.Arg647∗	14	This study	UFS (grimace and urinary tract disease)
c.1948C>T	Misssense variant p.Arg650Cys	14	Roberts *et al.*^21^	Urinary tract disease (without UFS grimace)
c.1980_1981ins371 GenBank JX891452 and c.2088delC	Frameshift variant p.Ile662fs and Stop variant p. Ser697Hisfs∗11	14	Stuart *et al.*^17^	UFS (grimace and urinary tract disease)
c.2125C>T	Stop variant p.Arg709∗	15	Stuart *et al.*^17^	UFS (grimace and urinary tract disease)
c.2125C>T	Stop variant p.Arg709∗	15	Fadda *et al.*^33^	Urinary tract disease (without classic UFS grimace but with other facial dysmorphology)
c.2554A>T	Misssense variant p.Ile852Phe	16	Stuart *et al.*^17^	Urinary tract disease (without UFS grimace)

Frameshift and stop variants have been associated with full-blown UFS, comprising of UT disease and a grimace on smiling. In contrast, missense variants are associated with UT disease without the grimace. All cases have homozygous variants apart from one compound heterozygous case, as indicated.

### General Characteristics of Lrig2 Mutant Mice and Their Bladders

To learn more about the underlying pathophysiology of *LRIG2* UFS, we studied *Lrig2* homozygous Mut mice. Given potential differences between the sexes, male and female mice were analyzed separately. Juvenile males were studied between 14 and 19 days after birth, with no significant difference in age between WT and Mut groups (Supplementary Figure S1A). Juvenile females were studied between 14 and 20 days, with no significant difference in age between WT and Mut groups (Supplementary Figure S1B). Male Mut mice weighed significantly less than their WT littermates (Supplementary Figure S1C), and female Mut mice weighed significantly less than WT (Supplementary Figure S1D). Inspection on autopsy indicated that in each sex, Mut bladders were more prominent than WT bladders, with Mut bladders typically distended with urine (Supplementary Figure S2A), as previously reported.^21^ There was no significant difference between the 2 genotypes in the weights of male bladders (body only, without outflow tract) drained of urine (Supplementary Figure S1E) but the bladder-to-body weight ratio (Supplementary Figure S2B) was significantly higher in male mutants. The weight of female bladders (body only, without outflow tract) drained of urine was greater in the Mut versus WTs (Supplementary Figure S1F). Female bladder-to-body weight ratio (Supplementary Figure S2B) was also higher in Mut mice.

### Bladder Histology

**Figure 2:**
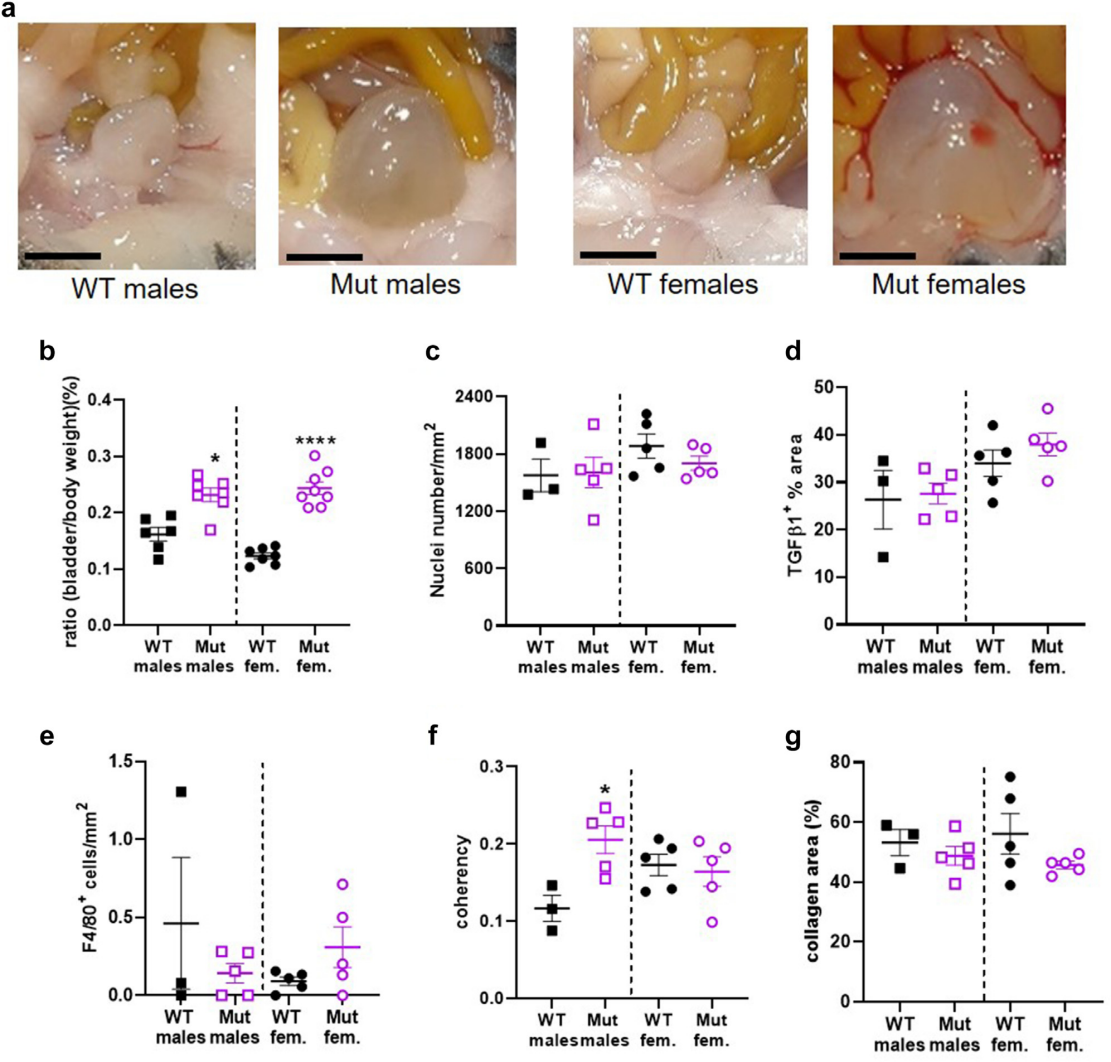
WT and *Lrig2* Mut bladders. (a) Representative bladders at autopsy. Note the Mut bladders were distended with urine. Scale bars are 2 mm. (b) Bladder-to-body weight ratio was higher in Mut versus WT males (WT *n* = 6, Mut *n* = 7, *P* = 0.0018), and also in Mut *versus* WT female littermates (WT *n* = 6, Mut *n* = 8, *P* < 0.0001). (c) Nuclei number per area in the detrusor was similar between WT and mutant males (WT *n* = 3, Mut *n* = 5, *P* = 0.905), and between WT and mutant females (WT *n* = 5, Mut *n* = 5, *P* = 0.2506). (d) Percentage area positive for TGFβ1 staining in the detrusor was not different between male WT and mutant mice (WT *n* = 3, Mut *n* = 5, *P* = 0.819). It was also similar between female WT and mutant (WT *n* = 5, Mut *n* = 5, *P* = 0.315). (e) Cell numbers positive for the macrophage marker F4/80 in the detrusor were similar between male WT and mutants (WT *n* = 3, Mut *n* = 5, *P* = 0.3569) and between female WT and mutants (WT *n* = 5, Mut *n* = 5, *P* = 0.140). (f) Coherence of collagen fibrils in the detrusor layer was higher in male Mut compared with WT (WT *n* = 3, Mut *n* = 5, *p* = 0.0204) but it was similar between female WT and mutants (WT *n* = 5, Mut *n* = 5, *P* = 0.7256). (g) The percentage area positive for collagen staining in the detrusor was similar between male WT and mutants (WT *n* = 3, Mut *n* = 5, *P* = 0.434) but was similar between female WT and mutants (WT *n* = 5, Mut *n* = 5, *P* = 0.168). In this and in subsequent figures, WT males are represented by black filled squares, Mut males by purple open squares, WT females by black filled circles, and Mut females with purple open circles. Results are expressed as mean±SEM. ∗*P* < 0.05, ∗∗*P* < 0.01, ∗∗∗∗*P* < 0.0001 are the significant differences between Mut and WT of the same sex. WT, wild-type.

Given the enlargement of Mut bladders, we investigated histologic differences between Mut and WT bladders. Bladder domes sectioned and stained with hematoxylin had similar general appearances (Supplementary Figure S2A). The number of detrusor nuclei per area did not differ between the genotypes in males or in females (Figure 2c), suggesting a lack of significant cell hypertrophy. To determine whether inflammation or fibrosis might be present, TGFβ1, macrophages, and collagen were assessed. The percentage of detrusor area immunostaining for TGFβ1 (Supplementary Figure S2B) was similar (Figure 2d) between the genotypes in males and females. The number of macrophages in the detrusor (Supplementary Figure S2C and E) was also similar in the 2 genotypes. From picrosirius red staining and bright field imaging (Supplementary Figure S3A**)**, the percentage area positive for collagen in detrusor was similar between the genotypes (Figure 2g) for both males and females. Picrosirius red was also imaged under polarized light (Supplementary Figure S3C and D) to visualize birefringent collagen and then calculate the orientation or coherence of collagen fibrils. In the detrusor layer (i.e., smooth muscle bundles plus interstitial tissues between them), fibril coherence was significantly higher in *Lrig2* mutants; however, but no difference was found between genotypes in females (Figure 2f**).** With respect to the mucosa (i.e., urothelium plus lamina propria), coherence was similar in sex-matched mutants and wild-types (Supplementary Figure S3B)**.**

### Physiology of Isolated Bladder Outflow Tracts

**Figure 3:**
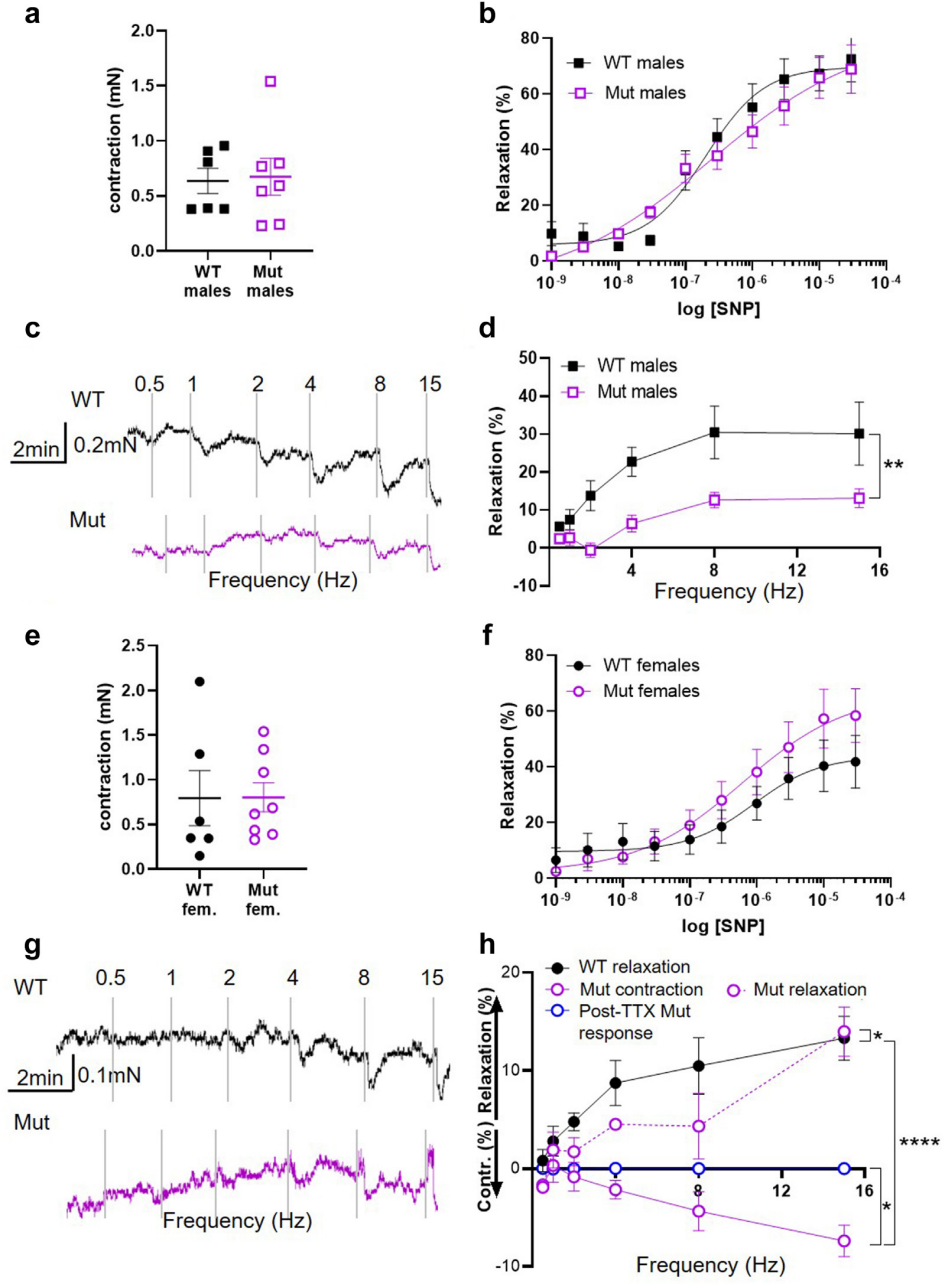
Dysfunctional outflow physiology in *Lrig2* mutant mice. (a) Amplitudes of contractions evoked by 50 mM KCl in male WT (*n* = 6) and Mut (*n* = 7) outflow tracts. (b) Outflow relaxations in response to cumulatively increased concentrations of SNP in WT (*n* = 6) and mutant (*n* = 6) male outflow tracts. Curves show best fits to the Hill equation with EC_50_ = 0.20 μM (control) and 0.24 μM (Mut) and E_max_ = 81.7 % (control) and 80.6 % (Mut). (c) Representative traces of WT and mutant male outflows contracted with 5 μM PE then stimulated by EFS at the frequencies indicated. (d) Mean ± SEM relaxations evoked by EFS, plotted as a function of frequency in male WT (*n* = 5) and Mut (*n* = 7) outflows; ∗∗*P* < 0.01. (e) Amplitudes of contraction evoked by 50 mM KCl in female outflows (WT *n* = 6, Mut *n* = 8). (f) Female outflow relaxations in response to cumulatively increased concentrations of SNP in WT (*n* = 6) and mutant (*n* = 8) outflow tracts. Curves show best fits to the Hill equation with EC_50_ = 0.91 μM (control) and 0.61 μM (Mut) and E_max_ = 43.7 % (control) and 66.5 % (Mut). (g) Representative traces of WT and mutant female outflows contracted with 10 nM AVP then stimulated by EFS at the frequencies indicated. (h) Mean responses to EFS as a function of frequency in female outflows (WT *n* = 6, Mut *n* = 8). Mutant contraction and relaxation were plotted separately. Responses of Mut outflows tested in the presence of tetrodotoxin (*N* = 4) are also shown. Results are expressed as mean ± SEM. ∗*P* = 0.02 Mut versus tetrodotoxin, ∗∗∗∗*P* < 0.0001 WT compared with Mut. WT, wild-type.

Having established that the Mut bladders were not overtly fibrotic or inflamed, we proceeded to undertake *ex vivo* physiology experiments on bladder outflow tracts using myography. In males, no significant difference in contraction amplitude to 50 mM KCl between Mut and WT tissues was observed (Figure 3a). As shown in Supplementary Figure S4A, PE-evoked contractions of male Mut outflows with similar potency and efficacy to WT preparations. However, after adjusting for KCl, there was a statistically significantly higher dose-response curve in mutants (Supplementary Figure S4B). The relaxation response to the NO donor SNP (Figure 3b) was also similar in Mut and WT outflow tracts. In contrast, EFS evoked frequency-dependent relaxation of PE-contracted outflows that were significantly smaller across all frequencies in Mut tissues compared to WT (Figure 3c and d). In addition, we found an overall similar pattern in adult (8–12-week-old) male outflow tracts, although the EFS-induced relaxation deficit was more pronounced at lower frequencies, converging at 15 Hz (Supplementary Figure S5A–E). Outflow tracts from females with Mut and WT genotypes had similar amplitude contractions in response to 50 mM KCl (Figure 3e) and relaxed with similar responses to SNP (Figure 3f). A striking difference was observed, however, in their responses to EFS (Figure 3g and h). Female WT outflows precontracted with AVP displayed frequency-dependent relaxation in response to EFS, although the responses were smaller than observed in males. In Mut outflows, EFS evoked frequency-dependent contraction, which was followed by a small relaxation. Contractions and relaxations at all frequencies were blocked by preincubating Mut outflows with tetrodotoxin, which prevents neurotransmission in the tissue by blocking Na channels.

### Physiology of *Ex Vivo* Bladder Body Preparations

**Figure 4:**
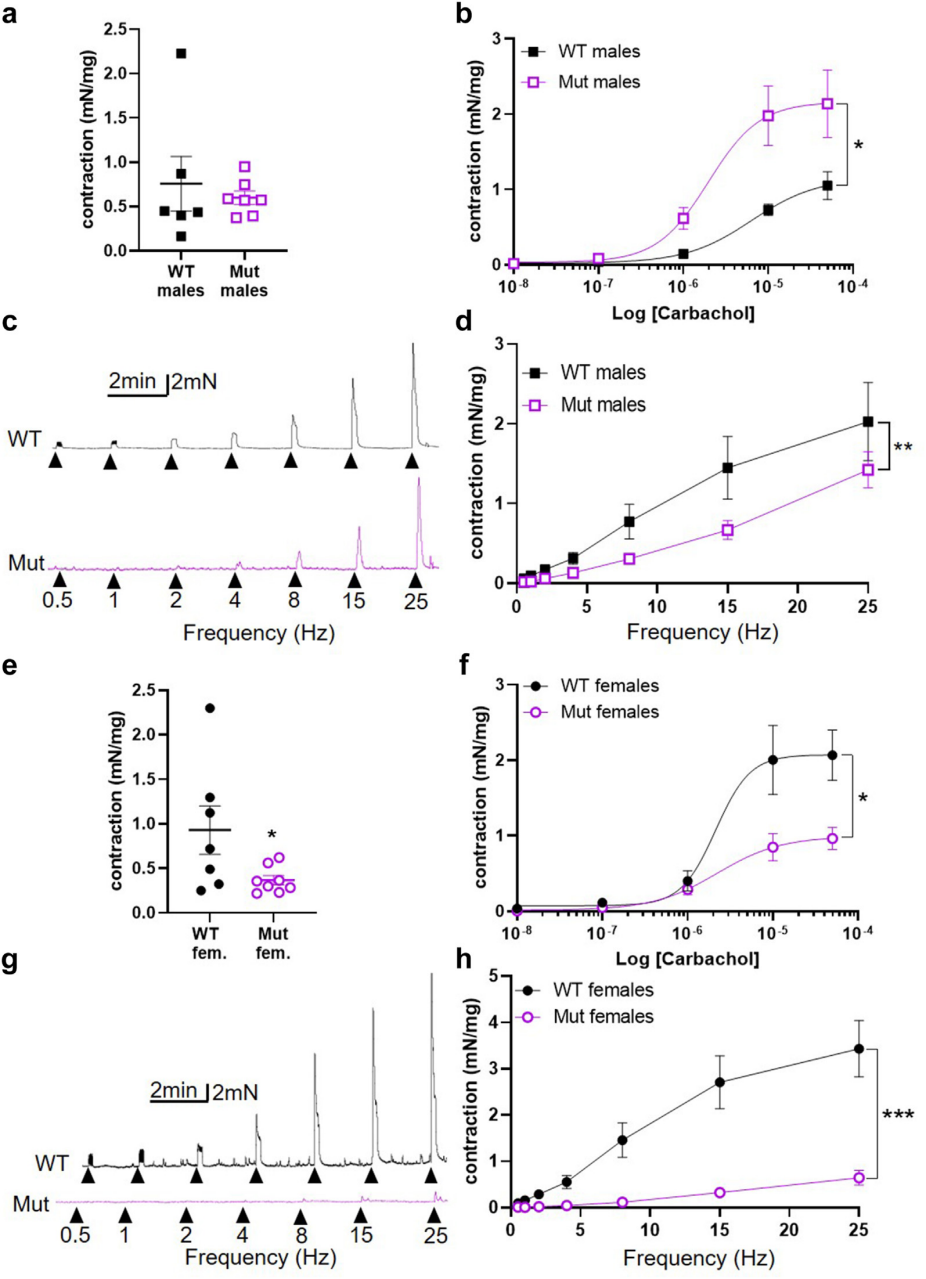
Dysfunctional bladder body physiology in *Lrig2* mutant mice. (a) Amplitudes of contractions evoked by 50 mM KCl in bladder rings from WT (*n* = 6) and Mut (*n* = 7) male mice. (b) Contraction of bladder rings from WT (*n* = 5) and Mut (*n* = 7) male mice in response to cumulative application of 10 nM – 50 μM carbachol, plotted as a function of carbachol concentration. Curves are the best fits of the Hill equation with EC_50_ = 6.37 μM and E_max_ = 1.15 mN/mg in WT mice compared with EC_50_ = 1.97 μM and E_max_ = 2.16 mN/mg in Mut mice. ∗*P* = 0.04 comparing WT and Mut by 2-way analysis of variance with repeated measures (c) Representative traces of contraction evoked in male WT and Mut bladder rings in response to EFS at the frequencies indicated. (d) Mean amplitudes of contraction evoked by EFS in bladders from male WT (*n* = 5) and Mut (*n* = 7) mice plotted as a function of frequency; ∗∗*P* = 0.0075. (e) Amplitudes of contractions evoked by 50 mM KCl in bladder rings from WT (*n* = 7) and Mut (*n* = 8) female mice. (f) Contraction of bladder rings from WT (*n* = 7) and Mut (*n* = 8) female mice in response to cumulative application of 10 nM – 50 μM carbachol, plotted as a function of carbachol concentration. Curves are the best fits of the Hill equation with EC_50_ = 2.13 μM and E_max_ = 2.07 mN/mg in WT mice compared with EC_50_ = 2.26 μM and E_max_ = 0.99 mN/mg in Mut mice. ∗*P* = 0.02 comparing WT with Mut by 2-way ANOVA. (g) Representative traces of contraction evoked in female WT and Mut bladder rings in response to EFS at the frequencies indicated. (h) Mean amplitudes of contraction evoked by EFS in bladders from female WT (*n* = 5) and Mut (*n* = 7) mice plotted as a function of frequency. ∗∗∗*P* = 0.0006 comparing WT and Mut by 2-way ANOVA with repeated measures. Results are expressed as mean ± SEM. WT, wild-type.

Rings of bladder bodies from Mut and WT male mice contracted equally in response to the application of 50 mM KCl (Figure 4a). In contrast, bladders from Mut male mice were more sensitive to carbachol than those from WT males (Figure 4b), with a significantly higher dose-response curve to carbachol. Bladder rings from male mice contracted in response to EFS, the contraction increasing in amplitude with stimulation frequency (Figure 4c). Despite their increased responsiveness to carbachol, responses to EFS were statistically significantly smaller in bladders from Mut mice compared with WT mice (Figure 4d). In addition, we found an overall similar abnormal pattern in adult (8–12 weeks) male bladders (Supplementary Figure S5F–H). Bladder rings from female Mut mice contracted less forcefully to 50 mM KCl than rings from WT females (Figure 4e). Rings of Mut female bladder also contracted less to carbachol than WT female bladder, in Mut mice (Figure 4f), with a significantly lower dose-response curve to carbachol. EFS evoked frequency-dependent contraction in bladder rings from female WT and Mut mice, but the responses of Mut bladders were much weaker with the force-frequency curve lower than could accounted for by reduced myogenic power alone (Figure 4g and h).

## Discussion

This study advances our understanding of the genetics and pathophysiology of early onset bladder disease, combining insights from human disease and a mouse model. We place the current *LRIG2* variant in the context of the stop or frameshift *LRIG2* variants previously reported in UFS. In addition, we point out that biallelic missense *LRIG2* variants lead to UT-limited disease (i.e., affected people lack the facial grimace found in the complete UFS syndrome). The current *ex vivo* physiology experiments complement our initial published study,^21^ in which we reported that LRIG2 is immunodetected in pelvic ganglia (that send autonomic axons into the bladder) of healthy mice; and *Lrig2* mutant mice have abnormal urination patterns (like people with UFS), as well as abnormally patterned bladder nerves (as assessed by whole-mount peripherin immunostaining). The novel mouse studies we report here, which point to mainly neurogenic functional defects, support the overall idea that biallelic *LRIG2* variants lead to human bladder disease through autonomic neural defects that perturb bladder function.

### Insights Into LRIG2 Associated Human Bladder Disease

We describe a variant in *LRIG2* in a hitherto unreported family with UFS. The variant in exon 14 results in a stop codon in the second immunoglobulin-like domain of the extracellular part of the protein. Putting this new family in the context of all reported UT-disease associated *LRIG2* variants, as depicted in Figure 1d and Table 1, it is evident that biallelic stop or frame shift variants, consistent with a putative loss of function mechanism, can be associated with a complete UFS phenotype, combining the characteristic grimace with UT disease, as reported in this and other^17^^,^^18^ studies. This relationship is not exact because one study reported a stop variant with marked UT disease but without the typical inverted smile^33^; that individual, however, did have an unusual smile and other facial dysmorphology. In contrast, individuals with biallelic missense variants^17^^,^^21^ uniformly lack the UFS facial grimace but have severe functional bladder outlet obstruction. Further research is needed to explain these genotype-phenotype relationships. However, the unified message to clinicians is to consider *LRIG2* and *HPSE2* variants as a cause of congenital bladder obstruction whether a UFS grimace is present or not. The patient we describe in the current report was exposed to gestational diabetes mellitus. Human clinical studies as well as animal experiments suggest that maternal diabetes mellitus can perturb UT development.^34^, ^35^, ^36^ The phenotypes associated with maternal diabetes, however, are kidney hypoplasia or agenesis rather than functional bladder outflow obstruction. Therefore, the UT phenotype in the current case is highly unlikely to have been modified by maternal diabetes.

### Initial Evidence That a Peripheral Neuropathy Underlies UFS

Individuals with UFS usually present with dysfunctional urinary voiding and urosepsis in early childhood,^12^^,^^13^ and it is also recognized that some such patients have large bladders visualized before birth.^14^ Indeed, the individual described in this study had all of these features. Key questions are, therefore, to understand the biologic and physiologic roles of *LRIG2* and *HPSE2* in healthy bladders, and how these processes go wrong in the presence of germline variants to generate the clinical phenotypes. LRIG2 and heparanase-2 proteins have been detected in nerves within the late first trimester in human fetal bladder,^17^ and in their migrating neural crest precursors in earlier human embryos.^11^ Moreover, both proteins are present in mouse pelvic ganglia,^20^^,^^21^ which send postganglionic autonomic neurons into the lower UT.^21^^,^^37^ Homozygous *Lrig2* or *Hpse2* mutant mice both have abnormally patterned bladder nerves.^21^ Full length LRIG2 is a plasma membrane protein but a soluble cleaved form has also been described.^38^ LRIG2 enhances tumor growth in diverse tissues, including brain and skin, a property conferred in part by modulating growth factor signaling.^22^^,^^39^^,^^40^ Mouse studies have also implicated LRIG2 in axon guidance during central nervous system development and regeneration.^41^ Collectively, these observations led us to predict that LRIG2 has important roles in the physiology of urinary voiding, and we hypothesized that peripheral neurogenic defects are present in *LRIG2*-associated bladder dysfunction.

### Lrig2 Mutant Mice Have Neurogenic Defects in Bladder Outflow Tract Relaxation

For optimal voiding to occur, the outflow tract must relax when the bladder body contracts. NO released from nitrergic nerves is considered the primary mechanism of outflow tract relaxation during micturition in mammals.^24^ In male *Lrig2* mutant mice, we observed reduced nitrergic-nerve mediated outflow relaxation. The outflow maintained its responsiveness to SNP, implying that the loss of nitrergic relaxation in the mutant outflow was due to less NO reaching the muscle rather than an altered NO action. This accords with a report that *Lrig2* mutant mouse outflow tracts appear to contain fewer nitrergic nerves than WT controls.^21^ Male *Lrig2* mutant outflows were otherwise “physiologically normal.” Contractions induced by KCl or the α1-receptor agonist PE were preserved in mutant outflow tracts, again indicating that nonneurogenic contraction of the outflow smooth muscle is normal in *Lrig2* Mut mice. Cautiously extrapolating to humans, male UFS patients might be predicted to have a functional bladder outflow obstruction with an important neurogenic component, specifically with diminished nitrergic-nerve function. Interestingly, when adjusted for KCl, male outflow tract contractile responses to PE were greater in mutants than controls, suggesting that postsynaptic functions in the smooth muscle of the bladder outflow tract may also be altered. Given that LRIG2 is normally detected in bladder nerves rather than SM,^21^ this effect may be secondary to neural defects.

Female *Lrig2* mutant mouse outflow tracts differed in detail from the male mutants, showing a more dramatic loss of nitrergic relaxation, yet the functional effect would be the same, that is, bladder outflow obstruction. Indeed, reviewing the collective but limited literature (the current study;^17^^,^^18^) males and females with UFS and *LRIG2* variants have UT disease of apparently similar severity. We used a modified protocol to investigate neurogenic function in female mice, because WT female outflows did not contract to α_1_-receptor agonists, possibly because of the absence of sympathetic nerves, neurotransmitter release, or receptors. Female outflow tracts were instead contracted with AVP to enable relaxation to be studied, as previously described.^27^ In that context, EFS evoked relaxation responses that were frequency-dependent and tetrodotoxin-sensitive in female WT outflow tracts, as seen in males.^23^ In addition to reduced relaxation, EFS further evoked an initial paradoxical contraction of *Lrig2* mutant female outflows precontracted with AVP, which was absent in WT preparations. These contractions were also tetrodotoxin-sensitive, indicating a neuronal origin but we were unable to identify the neurotransmitter involved. The impaired relaxation and paradoxical contraction in the mutant female outflows would result in impaired urination *in vivo*, which appears consistent with the functional bladder outflow obstruction reported in people with UFS.^12^ However, we must be cautious about the detailed mechanistic similarities between mice and humans. Female neurourological anatomy and function can vary between species. Although the outflow tracts of female mice lack a sympathetic contractile response, this may not be typical of larger mammals. For example, α_1_-receptor stimulation induced muscle contraction in urethral preparations from female pigs^42^ and humans.^43^ Moreover, similar levels of α_1_-receptor transcripts were detected in female and male outflow tracts from patients with invasive bladder cancer.^44^ Pigs may represent an important model for studying the neurophysiology of human female outflow tracts. In future it may be possible to model UFS in pigs by knocking down the implicated genes, as has been done for spinal muscular atrophy.^45^ These considerations also point to the need for further work to test the physiology of bladder outflow tracts in healthy male and female humans, and in individuals with UFS. A working hypothesis is that in both males and females with UFS, there is defective urethral dilation with differing underlying pathophysiology. If this is correct, then different types of drug may be optimal to help bladder emptying in males or females with UFS.

### Lrig2 Mice Also Have Abnormal Detrusor Contractility

Our investigation of the bladder body of *Lrig2* mutant mice also revealed a strong neurogenic phenotype. Frequency-dependent contractions evoked by EFS were smaller in the mutant bladder preparations, with the difference between WT and mutant more pronounced in females than males. These findings are broadly consistent with a previous study of juvenile male *Hpse2* mutant mice.^23^ There is an increase in nerve density (as assessed by peripherin whole-mount immunostaining) in the bladder body of juvenile *Lrig2* (and *Hpse2*) mutant mice compared with control littermates.^21^ Our new data suggest that despite this increase, mutant nerves are unable to elicit normal contractions. Further, in male mutant bladders the reduced contraction force began to converge with WT at higher frequencies, suggesting that neurogenic stimulation is capable of driving WT-level contraction, but only with much greater stimulation. The exact mechanism behind this, and how cholinergic and purinergic signaling are affected, will be the subject of future pharmacologic studies. Effects of the *Lrig2* mutation on muscle contraction evoked directly by membrane depolarization or receptor stimulation also diverged between the sexes. The male *Lrig2* mutant bladder generated a normal level of contraction in response to depolarization with KCl but was hyper-responsive to muscarinic receptor-mediated stimulation. In female mice, contractile responses to both KCl and muscarinic receptor stimulation were suppressed in the *Lrig*2 mutant bladder body. The muscarinic M3 receptor is the predominant mediator of muscarinic stimulation in mouse and human bladder bodies^46^, ^47^, ^48^ and is activated by acetylcholine released from parasympathetic nerves. This activates G-proteins and their downstream signaling cascades to induce bladder contraction. The hypersensitivity to carbachol in male Mut mice could be due to upregulation of this pathway, rather than a general increase in contractility, because it was not seen with KCl-induced contraction. It could be a primary mechanism of UFS pathology, or a secondary response to the loss of parasympathetic input. Further work is needed to assess these possibilities. Whatever the mechanism, it was not seen in females, which rather showed a general loss of detrusor power, analogous to bladder decompensation reported in long-term diabetes mellitus, as reviewed.^29^

The apparently more severe *ex vivo* physiological defects in female *Lrig2* mutant bladder bodies, compared with males, correlates with the more marked change in bladder weight found in female mutants. Although in both sexes the bladder-to-whole body weight ratios were increased compared to WT mice, only the female mutants had an absolute increase in bladder weight compared with the WT controls. This difference may be a primary feature of the disease or, perhaps more likely, it reflects a more marked growth response to outflow obstruction in females than was present in males. In general, differences between males and females in their physiological responses could be a direct consequence of the mutation in *Lrig*2, or they could occur secondary to outflow obstruction. The current study could not address this question, but we note that there was no evidence of a fibrotic or inflammatory process in the detrusor smooth muscle of either sex on histology. The only difference found between mutants and controls was an increased collagen coherence in the detrusor layer of male mutants compared with male controls. We speculate that this may reflect higher hydrostatic pressures within these bladders, by analogy the relation between intraluminal pressure and collagen coherence in (nonmutant) arteries.^49^ It is of interest that patients with UFS have been recorded with raised hydrostatic intravesical pressures^12^ and intravesical pressures are raised in juvenile *Hpse2* mutant mice^50^ In the future, the current *ex vivo* physiology studies in *Lrig2* mutant mice could be complemented by performing *in vivo* experiments measuring bladder-urethra reflexes^51^ and intravesical pressures.^50^

### Lrig2 Mutant Bladder Defects Persist to Adulthood

We undertook another set of experiments with the focus on adult (8–12 weeks old) male mice. We show that these older *Lrig2* mutant animals have broadly similar *ex vivo* physiological defects in the bladder outflow and body as seen in juveniles. This allows us to make the important conclusions that the defects in juveniles cannot be simply explained away as delayed maturation but instead represent more fundamental neural functional aberrations that do not correct themselves with age. Future *ex vivo* physiological experiments on neonatal and even late fetal mutant mice will be needed to establish the exact onset of these defects. In fact, we have previously reported that neonatal *Lrig2* mutant bladder display deregulated levels of transcripts, as assessed by RNA sequencing, implicated in neural differentiation.^21^

### Future Perspectives

An important avenue of future study will be to investigate the dyssynergia between bladder and outflow using *in vivo* cystometry with combined bladder and outflow monitoring.^51^ Such experiments in *Lrig2* mutant mice will address endogenous defects in the bladder body and outflow interact to cause a functional dyssynergia. As pointed out earlier in this study, people with UFS not only have urination defects but are at risk of ascending bacterial urosepsis that can damage the kidneys. Our previous study^21^ showed that kidney histology appears normal in juvenile *Lrig2* mutant mice, so we did not repeat this investigation in the current study. In our animal facility, the *Lrig2* mutant mice are bred and maintained in a clean environment, and they do not appear to suffer from spontaneous urosepsis in this setting.

### Conclusions

This study highlights several notable clinical, genetic, and pathobiological points. The current case constitutes further evidence that biallelic *LRIG2* variants can cause UFS. It also emphasizes that UT disease in UFS begins before birth. Putting this family in the context of all reported UT disease-associated *LRIG2* variants reported, full-blown UFS occurs with loss of function variants, but missense variants lead to bladder-limited disease without the grimace. For the clinician, it is important to consider *LRIG2* and *HPSE2* variants as a cause of congenital bladder obstruction whether a UFS grimace is present or not. Moreover, our murine observations support the idea that UFS is a genetic autonomic neuropathy of the bladder affecting functionality of the outflow tract and the bladder body. The nuanced sex differences in abnormal *ex vivo* physiology in *Lrig2* mutant mice may suggest that drug treatments could be tailored for males or females with UFS and should prompt further studies of possible sex differences in bladder outflow and body physiology in humans.

## Disclosure

All the authors declared no competing interests.

## Supplementary Material

**Supplementary Methods.**

**Figure S1.** Mouse ages and bladder weights**.**

**Figure S2.** Histology of WT and *Lrig2* Mut male and female bladders**.**

**Figure S3.** Picrosirius red staining of bladder dome histology sections**.**

**Figure S4.** Male outflow responses to phenylephrine**.**

**Figure S5.** Adult male *ex vivo* physiology of the bladder outflow tract and body**.**
